# Convergence and Extrusion Are Required for Normal Fusion of the Mammalian Secondary Palate

**DOI:** 10.1371/journal.pbio.1002122

**Published:** 2015-04-07

**Authors:** Seungil Kim, Ace E. Lewis, Vivek Singh, Xuefei Ma, Robert Adelstein, Jeffrey O. Bush

**Affiliations:** 1 Department of Cell and Tissue Biology, Program in Craniofacial Biology and Institute for Human Genetics, University of California, San Francisco, California, United States of America; 2 Laboratory of Molecular Cardiology, National Heart, Lung, and Blood Institute, National Institutes of Health, Bethesda, Maryland, United States of America; California Institute of Technology, UNITED STATES

## Abstract

The fusion of two distinct prominences into one continuous structure is common during development and typically requires integration of two epithelia and subsequent removal of that intervening epithelium. Using confocal live imaging, we directly observed the cellular processes underlying tissue fusion, using the secondary palatal shelves as a model. We find that convergence of a multi-layered epithelium into a single-layer epithelium is an essential early step, driven by cell intercalation, and is concurrent to orthogonal cell displacement and epithelial cell extrusion. Functional studies in mice indicate that this process requires an actomyosin contractility pathway involving Rho kinase (ROCK) and myosin light chain kinase (MLCK), culminating in the activation of non-muscle myosin IIA (NMIIA). Together, these data indicate that actomyosin contractility drives cell intercalation and cell extrusion during palate fusion and suggest a general mechanism for tissue fusion in development.

## Abstract 2

A study of the mouse palate shows that the fusion of tissues during development involves convergence and displacement of epithelial cells, coupled with cell extrusion driven by the contractile activity of actomyosin.

## Author Summary

Tissue fusion, the process by which two independent prominences become united to form one continuous structure, is common during development, and its failure leads to multiple structural birth defects. In this study, we directly examine the cellular and molecular mechanisms by which tissue fusion occurs using the mouse secondary palate as a model. Using live imaging, we find that fusion of the secondary palatal shelves proceeds by a progression of previously undescribed cell behaviors. Cellular protrusions and establishment of contacts between palatal shelves leads to the formation of a transient multicellular epithelial structure that then converges toward the midline, driven by cell intercalation. This convergence occurs together with displacement of the epithelium and epithelial cell extrusions that squeeze epithelial cells out from between the palatal shelves and mediate continuity of the structure. We show that in mice this morphogenesis requires an actomyosin contractility pathway culminating in non-muscle myosin IIA activation. Altogether, these data support a new model for tissue fusion during mouse embryogenesis in which convergence, displacement, and cell extrusion drive the union of independent structures.

## Introduction

Tissue fusion is required for the morphogenesis of numerous vertebrate organ systems, including neural tube closure, heart morphogenesis, urogenital development, and craniofacial development, and failure of tissue fusion leads to birth defects in these contexts [1]. In craniofacial development, tissue fusion is required during the formation of the primary and secondary palates, with deficits in these processes resulting in cleft lip and cleft palate, respectively [2,3]. The secondary palate arises from bilateral outgrowths of the maxillary processes called palatal shelves, which undergo a highly coordinated morphogenesis involving vertical outgrowth, elevation, horizontal growth, and ultimately fusion with one another to form the intact roof of the mouth [4]. The external surface medial edge epithelium (MEE) of the palatal shelves is composed of an outer layer of flat periderm cells covering an inner layer of basal cuboidal cells on a basement membrane [5,6]. Periderm cells have been proposed to provide temporal and spatial regulation of adhesion competence and are thought to undergo apoptosis and slough off immediately prior to palatal shelf contact [5]. Electron microscopy studies of unpaired palatal shelves have shown that cells of the MEE extend filopodial and lamellipodial projections prior to the palatal shelves touching [7–9]. Whether projections persist until the shelves meet and whether they have functional significance in the initiation of fusion is not clear. Additionally, relatively little is known about the dynamic cellular behaviors that occur immediately upon contact of the independent palatal shelves. Static histological observations have indicated that apposing palatal shelf epithelial cells combine to form a common medial epithelial seam (MES), possibly by a convergent extension-like mechanism, but no direct evidence of convergence has been documented, and how these epithelia integrate is not known [10–12].

After the palatal shelves meet, the MES must be removed to achieve confluence of the underlying mesenchyme. The cellular mechanisms by which this occurs have been the subject of considerable investigation, and three mechanisms have been proposed: (1) epithelial to mesenchymal transition (EMT), (2) apoptotic cell death, and (3) cell migration [7,13]. Initial support for EMT, based on histological observation and ex vivo lineage tracing with vital dyes [5,14–16], has since been refuted by genetic lineage tracing experiments showing that the palatal epithelium does not give rise to mesenchymal cells that are maintained in the secondary palate [17,18]. Instead, it has been proposed that apoptotic cell death may be solely responsible for disappearance of the MES. Indeed, there have been multiple reports of apoptosis in the MES during fusion stages, and apoptosis is reduced in some mutants that fail to undergo proper palatal fusion [2]. Whether apoptosis is sufficient for removal of the MES is uncertain, however, because pharmacological or genetic inhibition of apoptosis has been inconclusive [19–24]. Finally, based on studies involving epithelial labeling and static observation at progressive time points, MES cells have also been proposed to actively migrate in the oronasal and anteroposterior dimensions to allow confluence of the underlying mesenchyme [10,23]. No direct observation of cell migration in the palatal epithelium has been reported, however, and relevance of migration in palate fusion remains unknown.

Cell migration and other morphogenetic cell behaviors are mediated by non-muscle myosin II (NMII), a subfamily of actin-based molecular motors that generate actomyosin contractility [25,26]. Each NMII unit is a hexamer composed of a pair of heavy chains (NMHC), a pair of essential light chains (ELC) and a pair of regulatory light chains (RLC). Three different isoforms of NMII (NMIIA, NMIIB, and NMIIC) are named according to the identity of their heavy chains, NMHCIIA, NMHCIIB, and NMHCIIC, which are encoded by the genes *Myh9*, *Myh10*, and *Myh14*, respectively. Whereas the ELC stabilizes the heavy chain structure, phosphorylation of the RLC positively regulates NMII-mediated actin contractility. Two major upstream kinases, rho-kinase (ROCK) and myosin light chain kinase (MLCK), phosphorylate RLCs to activate NMII [26,27].

Actomyosin contractility mediated by NMII has been demonstrated to be critical for a wide variety of morphogenetic events. During tissue closure events, such as *Drosophila* dorsal closure, *C*. *elegans* ventral enclosure, and Zebrafish epiboly, the formation of a supracellular actin cable at the leading edge of the epithelium maintains a uniform epithelial advance for which NMII provides contractile force to progressively close the opening in a purse-string or ratchet-like action [28–33]. During *Drosophila* dorsal closure, filopodia sample the closely apposed epithelium and begin to interdigitate, zipping the epithelia together [31,34]. Actomyosin contractility also drives intercalation and convergent extension behaviors during development. For example, NMII-driven actomyosin contractility drives cell neighbor exchange required for intercalation and germband extension in *Drosophila* and is required for polarized motility and convergent extension in *Xenopus* [35,36].

Here, we analyze the cellular mechanisms of tissue fusion in the mammalian secondary palate using live imaging to reveal the dynamic and multistep nature of this tissue fusion. We find that the formation and subsequent resolution of a multilayered epithelium to a shared, single-cell layer MES involves cell intercalation behaviors and convergence of the MES to the midline. Concomitant to this convergence, MES cells undergo coordinated cell movement in the oronasal axis and frequent cell extrusion events, indicating that convergent displacement and extrusion contributes to the removal of the MES. Actomyosin contractility is required for normal intercalation, displacement and extrusion of the MES cells, and disruption of actomyosin contractility by pharmacologic or genetic methods results in a failure of proper palatal shelf fusion. Perturbation of upstream regulators of actomyosin contractility had similar consequences on palatal shelf fusion, allowing the initial assembly of a pathway controlling these cell behaviors. These studies reveal a novel cellular mechanism for tissue fusion and provide a basis for studying the involvement of known regulators of tissue fusion in these cellular processes.

## Results

### Cell Intercalation and Oronasal Cell Displacement Are Prominent Cell Behaviors in the Fusing Palatal Shelves

**Fig 1:**
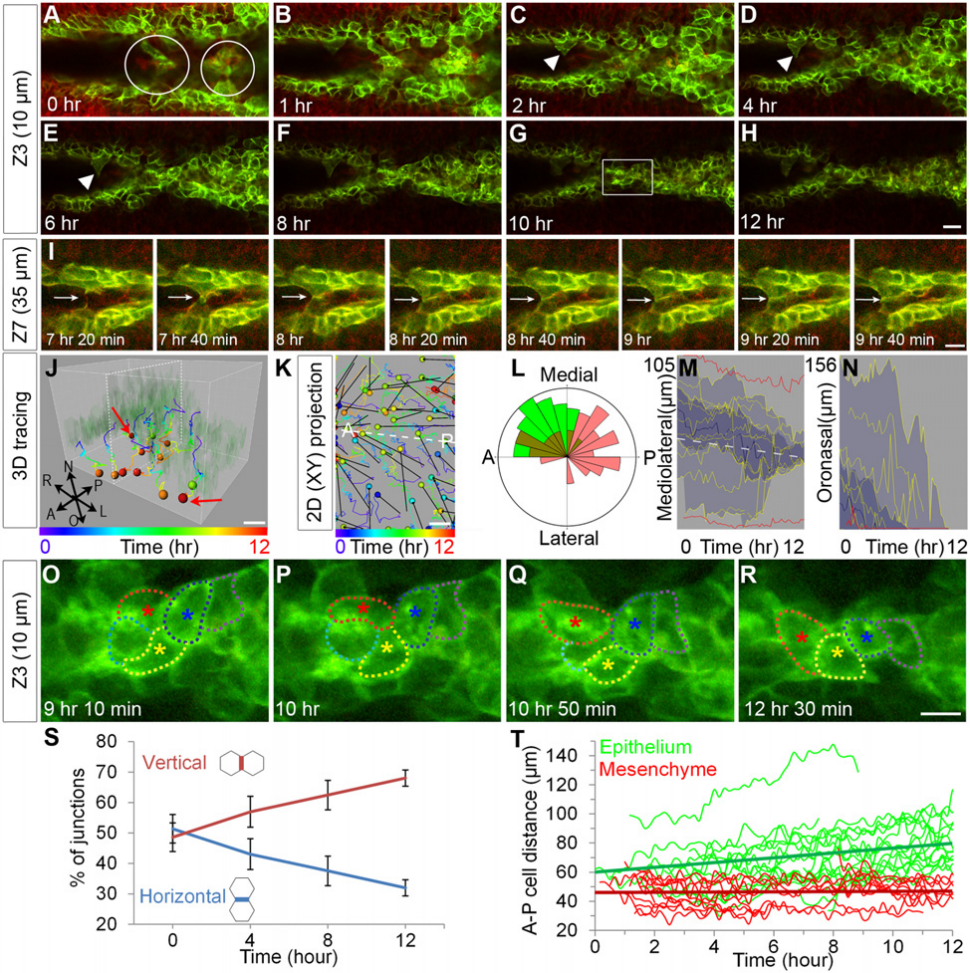
Cellular protrusions and cell intercalation during initiation of palatal fusion. (A–H) Confocal time-lapse imaging of *K14-Cre; ROSA26* ^*mTmG/+*^ palatal explant cultures reveals initial contacts made by cellular protrusions (white arrowhead) from each epithelium that form cellular bridges (white circles in A). Over the 12-hour imaging period, a multicellular epithelium becomes integrated into a single shared MES. Scale bar, 20 μm. (I) Higher magnification view of deeper optical sections reveals membrane extension and junction formation during palatal adhesion. Scale bar, 10 μm. (J) 3-D tracking and (K) 2-D tracking of cells in projection from all optical sections shows that epithelial cells in the MES converge toward the midline and displace toward the oral surface. The colors of the tracks indicate time; the colors of spheres indicate the last time points at which cells could be tracked. Dashed white lines in J and K indicate the plane of the MES. In K, displacement is shown with black arrows. Scale bar, 10 μm. A: anterior, P: posterior. (L) Rose diagram indicating angle of displacement for tracked cells (green, epithelium; red, mesenchyme) (M) Vantage plot of mediolateral position over time indicates that cells are converging to the midline. The approximate position of the MES midline is shown with a white dashed line. (N) Vantage plot of oronasal position over time indicates oral displacement of MES cells. In M, N the yellow lines are individual cell positions, the thick blue line is the median of the cell movements, the dark blue area is a range between the lower quartile and upper quartile, and light blue indicates the maximum range for 30 cells. Red lines are mediolateral (M) and oronasal (N) positions of lateral epithelial reference cells. (O–R) High magnification of time-lapse imaging in the region indicated in (G) outlines cells engaged in neighbor exchange. Scale bar, 10 μm. (S) Relative abundance of horizontal (-30°-30° to midline) and vertical (60°–120° to midline) junctions over the time course of palate fusion. Lines represent percentage of each junction type over *n* = 7 sections (relative mean ± SEM) (T) Distance between neighboring epithelial cells along the medial edge (green) or mesenchymal cells neighboring parallel to the medial edge (red). Linear best fit trend lines for the average distance are shown by straight green and red lines. Please see S1 Data for raw data.

To directly observe epithelial cell behavior during the course of tissue fusion, we used confocal live imaging of recently adhered E14.5 palatal shelves in explant culture (S1A Fig). Because our conditions for explant culture were different from those previously reported, we first confirmed complete fusion of the secondary palate by histology (S1B Fig). For resolution of the epithelium in the context of the secondary palate mesenchyme, we took advantage of the *ROSA26* ^*mTmG*^ reporter mouse, which expresses membrane-targeted tdTomato prior to Cre-mediated excision, and membrane-targeted GFP following recombination mediated by an epithelial-specific *Cre* (*K14-Cre*) (S1C Fig) [37,38]. At the initiation of the culture period, the palatal shelves had made contact and adhered in the middle palate, but not yet in the anterior region. We first focused on cell behaviors occurring immediately before and during initiation of secondary palate fusion by imaging a region of the anterior palate that had not made contact. In this region, we observed active epithelial projections prior to fusion (Fig 1A–1H, arrowheads; S1 Movie); these appeared as larger cellular protrusions into the oral cavity rather than as thin filopodial projections. Protrusions were observed to reach across and form junctions with MEE cells from the opposite palatal shelf (Fig 1I, arrow; S2 Movie). Upon contact of these extensions with cells from the apposing palatal shelf, cellular bridges formed connecting the two shelves (Fig 1A, circles; S1 Movie). These bridges, which initially appeared at spatial intervals, filled in with epithelium moving from deeper optical sections to form a multilayered epithelium shared between the two palatal shelves (Fig 1A–1D; S1 Movie). Over a period of 12 h, the multilayered epithelium converged toward the midline, ultimately resolving in a single-layered MES that was shared between the two palatal shelves (Fig 1E–1H; S1 Movie). Cell tracking of epithelial cells from either side of the multilayered MES indicated a net displacement of cells from lateral to medial, whereas the mesenchyme showed less directed movement toward the midline, supporting the overt observation of epithelial convergence (Fig 1J, 1K, 1L and 1M).

As neighbor exchange is a defining characteristic of cell intercalation, we examined whether similar rearrangements occurred during the integration of the MES. Cells initially sharing junctions with three or four cells rearranged to align along the midline, ultimately sharing junctions only with two neighboring cells (Fig 1O–1R, S3 Movie). Such junctional rearrangements were observed 56 times across seven adjacent optical sections over the course of imaging. We further quantified the extent of junctional rearrangement by counting all horizontal (-30°-30°) and vertical (60°–120°) junctions relative to the midline. At the beginning of the culture period there were similar numbers of horizontal and vertical junctions; the proportion of junctions that were horizontal decreased with time while the proportion of vertical junctions increased, supporting the widespread occurrence of junctional rearrangement in the MES during convergence (Fig 1S). A hallmark of intercalation is an increase in the distance between neighboring cells, as cells intercalate between them and drive them apart. We found that the distance between the cell centers of pairs of neighboring epithelial cells (*n* = 19 pairs) consistently increased, whereas the distance between pairs of neighboring mesenchymal cells (*n* = 16 pairs) did not tend to increase (Fig 1T). Together, these data demonstrate that palate fusion is initiated by formation of a multicellular epithelium that resolves to a shared single-layer epithelium by an active cellular convergence process that involves widespread cell intercalation of MES cells.

We next sought to determine whether cell migration might play a role in the removal of the MES. Four-dimensional cell tracking and displacement analysis revealed collective displacement of deeper (i.e., more nasal) MES cells toward the oral surface (Fig 1J and 1N) relative to the lateral palatal epithelium, which did not show marked movement. This oronasal displacement was a consistent behavior that was shared by the bulk of the MES cells analyzed (Fig 1J and 1N). In a few cases in the middle palate, we also observed a coherent migration of MES cells on the oral surface toward the posterior unfused part of the secondary palate (S4 Movie). Together, these data provide direct evidence for involvement of cell displacement in the removal of the MES during palatal fusion.

### Non-muscle Myosin Activity Is Critical for Palatal Fusion

Cell intercalation and cell migration require actomyosin contractility provided by non-muscle myosin activity [26,39]. The three non-muscle myosin isoforms are defined by their heavy chain subunits, which exhibit tissue-specific and developmentally regulated expression and function [26,40,41]. We therefore sought to evaluate the involvement of each of the non-muscle myosin isoforms in palate fusion by examining expression of the three heavy chain genes. *Myh9* exhibited elevated mRNA expression in the MEE and presumptive point of fusion between the nasal septum and palatal shelves immediately prior to and during fusion stages; immunostaining for NMHCIIA confirmed this expression (S2A and S2D–S2I Fig) [42]. *Myh10* was expressed broadly throughout the mesenchyme and epithelium, though at apparently lower levels in the epithelium than *Myh9*, and *Myh14* was not detected by in-situ hybridization (S2B and S2C Fig). To determine whether NMII might be involved in palate fusion, we first treated E13.5 palatal explant cultures with blebbistatin, a selective inhibitor of NMII ATPase activity [43]. After 72 h of culture, the MES had disappeared in nearly all control DMSO-treated palatal explants (mean fusion score = 3.92), whereas treatment with 10 μM blebbistatin resulted in failed fusion and maintenance of an intact MES in most sections analyzed (mean fusion score = 2.36) (S2J–S2L Fig). This was not a consequence of effects on cell proliferation, as there was no change in the percentage of Ki67^+^ cells in the epithelium or mesenchyme as assayed by immunostaining (S2M Fig). Based on the relative expression of the three isoforms, we hypothesized that NMHCIIA might play a dominant role in this context; siRNA knockdown of *Myh9* in explant culture mimicked the effects of blebbistatin, supporting this hypothesis (S2N–S2S Fig).

**Fig 2:**
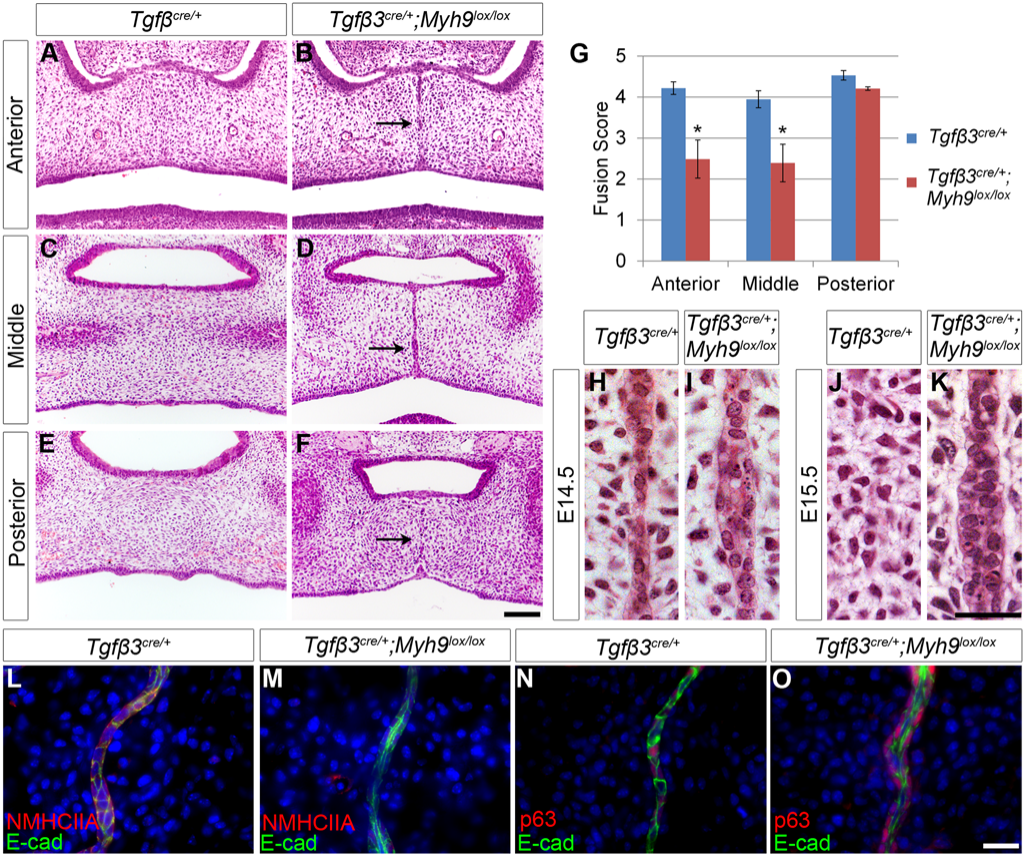
Epithelial loss of NMHCIIA causes defects in MES cell intercalation and MES persistence during palatal fusion. (A–K) *Myh9* deletion by *Tgfβ3* ^*cre/+*^ results in persistent MES cells upon loss of epithelial *Myh9* (B, D, F) compared with control (A, C, E) at E15.5. The observed defects were most severe in the anterior (B) and middle (D) regions of the palate. Scale bar, 100 μm. (G) Mean fusion scores were significantly reduced in *Tgfβ3* ^*cre/+*^;*Myh9* ^*lox/lox*^ palates compared with *Tgfβ3* ^*cre/+*^ controls. (H–K) High magnification images of histological sections of the MES region revealed that the midline MES is maintained as a multi-layered structure in *TgfB3* ^*Cre/+*^; *Myh9* ^*lox/lox*^ embryos at E14.5 (I) and E15.5 (K), while the control MES region has a thin MES at E14.5 (H) and no MES at E15.5 (J). Scale bar, 50 μm. (L, M) Immunostaining for NMHCIIA and E-cadherin indicates that NMHCIIA expression was lost in the multilayered MES of *Tgfβ3* ^*cre/+*^; *Myh9* ^*lox/lox*^ mutant E14.5 embryos (M) compared with control (L). (N, O) p63 expression was detected in the multilayered MES of *Tgfβ3* ^*cre/+*^;*Myh9* ^*lox/lox*^ mutant E14.5 embryos (O) as well as control embryos (N). In G, data are presented as mean fusion score ± SEM. *p < 0.05, Student’s *t* test, *n* = 3. Please see S1 Data for raw data.

We next tested whether genetic ablation of NMHCIIA function affected palatal fusion in vivo. Because generalized loss of function of NMHCIIA results in early embryonic lethality as a consequence of a failure to form a polarized visceral endoderm [44], we generated *Tgfβ3* ^*Cre/+*^; *Myh9* ^*lox/lox*^ embryos to mediate loss of NMHCIIA in the palate epithelium [45]. Whereas *Tgfβ3* ^*Cre/+*^ control embryos displayed a complete removal of the MES by E15.5, *Tgfβ3* ^*Cre/+*^; *Myh9* ^*lox/lox*^ embryos retained the MES at this stage (Fig 2A–2G). Because *Tgfβ3* ^*Cre*^ activity is not strictly restricted to the epithelium [45], we also employed *K14-Cre* to genetically ablate NMHCIIA from the fusing epithelium. Similar to *Tgfβ3* ^*Cre/+*^; *Myh9* ^*lox/lox*^ embryos, the MES at E15.5 failed to disappear in *K14-Cre; Myh9* ^*lox/lox*^ embryos (S3A Fig), supporting a specific requirement for NMHCIIA in the epithelium during palatal fusion. Greater dissolution of the MES occurred in the *K14-Cre; Myh9* ^*lox/lox*^ embryos compared with *Tgfβ3* ^*Cre/+*^; *Myh9* ^*lox/lox*^ embryos (S3B Fig). Though *Tgfβ3* ^*Cre*^ is known to mediate some recombination in the palate mesenchyme, cell proliferation index was not significantly different between *Tgfβ3* ^*Cre/+*^; *Myh9* ^*lox/lox*^ and control embryos, indicating that perturbed fusion was not attributable to effects of loss of NMHCIIA on mesenchymal cell proliferation (S3C Fig). Instead, this less severe phenotype corresponded to persistent higher levels of NMHCIIA expression in *K14-Cre; Myh9* ^*lox/lox*^ compared with *Tgfβ3* ^*Cre/+*^; *Myh9* ^*lox/lox*^ embryos, indicating that *K14-Cre* did not mediate complete loss of NMHCIIA from the MES (Fig 2L and 2M, S3D Fig). Examination of 100 histological sections across three embryos indicated that the MES defect in NMHCIIA-deficient embryos was consistent, and comparison of quantified fusion scores revealed significant differences between *K14-Cre; Myh9* ^*lox/lox*^ or *Tgfβ3* ^*Cre/+*^; *Myh9* ^*lox/lox*^ embryos and controls (Fig 2G; S3B Fig). When we examined the E14.5 MES at higher magnification we found that whereas control embryos already exhibited an integrated single-layered MES, *Tgfβ3* ^*Cre/+*^; *Myh9* ^*lox/lox*^ embryos maintained a multilayered epithelium that failed to converge (Fig 2H and 2I). Furthermore, at E15.5, when the MES was completely lost from control embryos, *Tgfβ3* ^*Cre/+*^; *Myh9* ^*lox/lox*^ embryos still retained a multilayered MES (Fig 2J and 2K). This multilayered epithelium, which could be visualized by E-cadherin immunostaining, was not composed solely of inappropriately retained periderm cells, because all cells appeared to express p63, a marker of the basal epithelium that is not expressed in periderm cells (Fig 2N and 2O) [46]. Failure of the normal removal of the MES did not lead to a cleft palate phenotype in *Tgfβ3* ^*Cre/+*^; *Myh9* ^*lox/lox*^ embryos, but the trapped epithelium was broken into smaller islands that were still apparent even at E17.5 (S3E Fig). Because *Myh10* exhibited broad expression that included the palate epithelium, we next asked whether the NMHCIIB isoform might also contribute to palatal fusion. Whereas disruption of NMHCIIB in *Tgfβ3* ^*Cre/+*^; *Myh10* ^*lox/lox*^ embryos did not lead to defects in MES removal (S3F Fig), *Tgfβ3* ^*Cre/+*^; *Myh9* ^*lox/lox*^; *Myh10* ^*lox/lox*^ mutant embryos did exhibit poorer MES removal and a submucous-type cleft of the posterior palate, supporting additive roles for NMHCIIA and NMHCIIB during palate fusion (S3G Fig). These results indicate that non-muscle myosin is critical for the organization of a multilayered epithelium into a shared MES and progression to normal fusion of the mammalian secondary palate.

### Upstream Regulators of Actomyosin Contractility Are Required for Palatal Fusion

**Fig 3:**
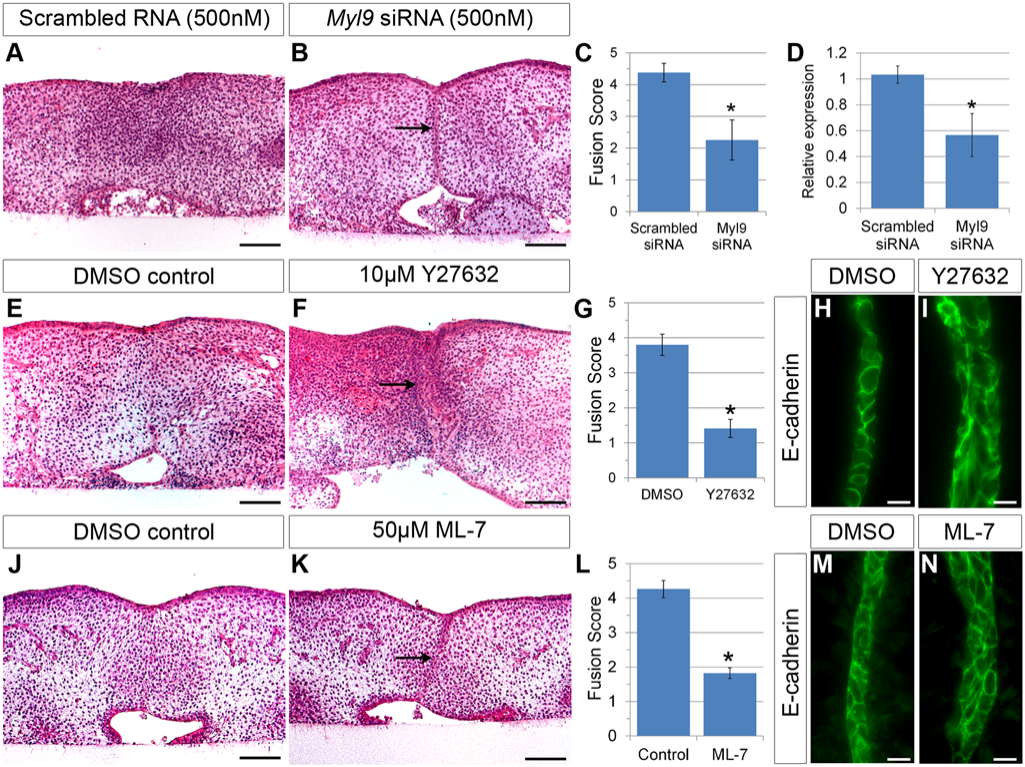
Inhibition of RLC *Myl9* or its upstream kinases ROCK or MLCK results in defective palatal fusion. (A–D) siRNA knockdown of *Myl9* resulted in significantly reduced palatal fusion (B, C) compared with control (scrambled RNA transfected) (A, C) in explants cultured for 72 h. Scale bar, 100 μm. (D) Verification of *Myl9* knockdown by siRNAs using qRT-PCR. *Myl9* siRNA treatments in explant culture decreased *Myl9* mRNA expression significantly. Student’s *t* test: * *p* < 0.05, *n* = 3. (E) Treatment with the ROCK inhibitor Y27632 blocked palate fusion (F, G) compared with DMSO control (E, G) in explants cultured for 72 h. Scale bar, 100 μm. (H, I) Immunostaining of E-cadherin in Y27632-treated explants cultured for 48 h illustrates maintenance of a multi-layered MES (I), whereas the control MES (H) has thinned by this stage. Scale bar, 20 μm. Treatment with the MLCK inhibitor ML-7 blocked palate fusion (K,L) compared with DMSO control (J,L) in explants cultured for 72 h. Scale bar, 100 μm. Immunostaining of E-cadherin in ML-7–treated explants cultured for 48 h showed maintenance of a multi-layered MES (N), whereas control MES had thinned by this stage (M).Scale bar, 20 μm. In (C, G, L), data are presented as mean fusion scores ± SEM. * *p* < 0.05, Student’s *t* test, *n* = 6 (C), *n* = 6 (G), *n* = 4 (L). Please see S1 Data for raw data.

NMII is regulated by phosphorylation of the regulatory light chain (RLC) by multiple kinases. For the most part, the context-specific expression and function of each of the six RLC genes has not been reported, though recently *Myl9* was shown to be involved in *Xenopus* convergent extension [47]. We used in-situ hybridization with probes against each of the RLC genes to determine that only one gene, the *Myl9* regulatory light chain gene, exhibited elevated expression in the fusing MES (S2T–S2V Fig). siRNA knockdown of *Myl9* led to significantly reduced palatal fusion after 72 h of explant culture (mean fusion score = 2.26) compared with scrambled siRNA control (mean fusion score = 4.38) (Fig 3A–3D). Rho kinase, ROCK, phosphorylates the RLC, and inhibitory myosin phosphatase, MYPT1 to activate actomyosin contractility during morphogenesis [48]. We therefore tested whether ROCK is involved in palatal fusion by pharmacologically inhibiting its function during palatal fusion. After 72 h in culture, DMSO-treated control palatal explants had fused (mean fusion score = 3.8), whereas treatment with the ROCK inhibitor Y27632 almost completely blocked palatal fusion (mean fusion score = 1.4), leading to the maintenance of a multi-layered epithelium (Fig 3E–3G). After 48 h of culture, DMSO-treated control explant cultures had established a single-layered MES, whereas Y27632-treated explant cultures failed to resolve the multilayered epithelium (Fig 3H and 3I), similar to what we observed in embryos lacking NMII function. Whereas ROCK has substrates independent of this pathway, myosin light chain kinase (MLCK) is relatively specific in its phosphorylation of the RLC to activate actomyosin contractility [26]. We therefore asked whether pharmacological inhibition of MLCK might also affect palatal fusion. Similar to ROCK inhibitor, treatment with the MLCK inhibitor ML-7 in 72 h explant cultures resulted in a significant reduction of palatal fusion (mean fusion score = 1.82) compared with control (mean fusion score = 4.26) (Fig 3J–3L); 48 h of culture in ML-7 resulted in a failure of the MES to integrate (Fig 3M and 3N), suggesting that MLCK may also be involved in this pathway.

**Fig 4:**
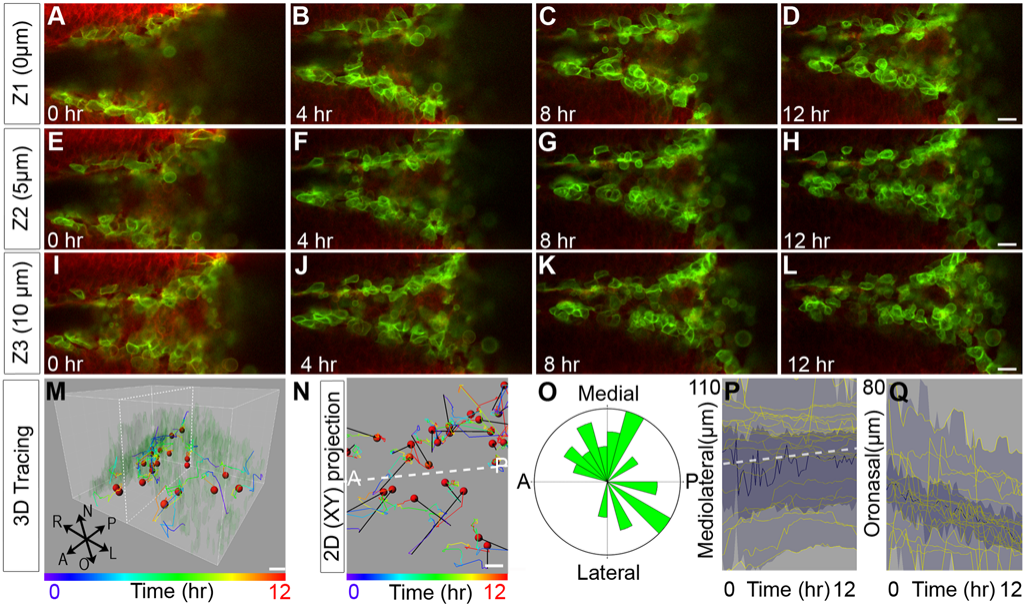
Confocal live imaging reveals a requirement for ROCK in MES intercalation and oral displacement. (A–L) Live confocal imaging of *K14-Cre; ROSA26* ^*mTmG/+*^ palatal explant cultures in the presence of 10 μM Y27632 at three different optical depths revealed defective convergence and no intercalation, with multiple layers of MES maintained after 12 h of culture. Scale bar, 20 μm. (M–N) 3-D (M) and 2-D (N) tracking shows considerably reduced oral displacement of the epithelium concomitant with failed convergence, though some random XY migration of MES cells was observed. Scale bar, 10 μm. Dashed white lines in M, N indicate the plane of the MES. (O) Rose diagram of angles of displacement of epithelial cells in explant culture treated with 10 μM Y27632. (P) Vantage plot of mediolateral position over time indicates that ROCK inhibition causes defects in cell convergence to the midline. (Q) Vantage plot of oronasal position over time indicates reduced oral displacement of MES cells. In (P, Q), the yellow lines are individual cell positions, the thick blue line is the median of the cell movements, the dark blue area is a range between the lower quartile and upper quartile, and light blue indicates the maximum range for 39 cells. Please see S1 Data for raw data.

Given the dramatic effect of ROCK inhibition on palatal fusion, we next asked how ROCK influences cell behaviors during the fusion process. Confocal live imaging of explant cultures treated with Y27632 revealed that although the epithelia of the palatal shelves became apposed, cell intercalation and neighbor exchange did not occur (Fig 4A–4L; S5 Movie). Cell tracking indicated that failure of cell intercalation was concomitant with a failure of the apposed epithelia to integrate toward the midline, and two layers of epithelium were still apparent even after 12 h of culture (Fig 4M–4P). Similarly, Y27632 prevented oronasal displacement of MES cells to the oral surface (Fig 4Q). Together these results indicate that an actomyosin contractility pathway including ROCK, MLCK, and NMII is required for the convergence and oronasal displacement behaviors underlying normal secondary palate fusion.

### Live Imaging of Actin Dynamics Reveals Contractility during Palate Fusion

**Fig 5:**
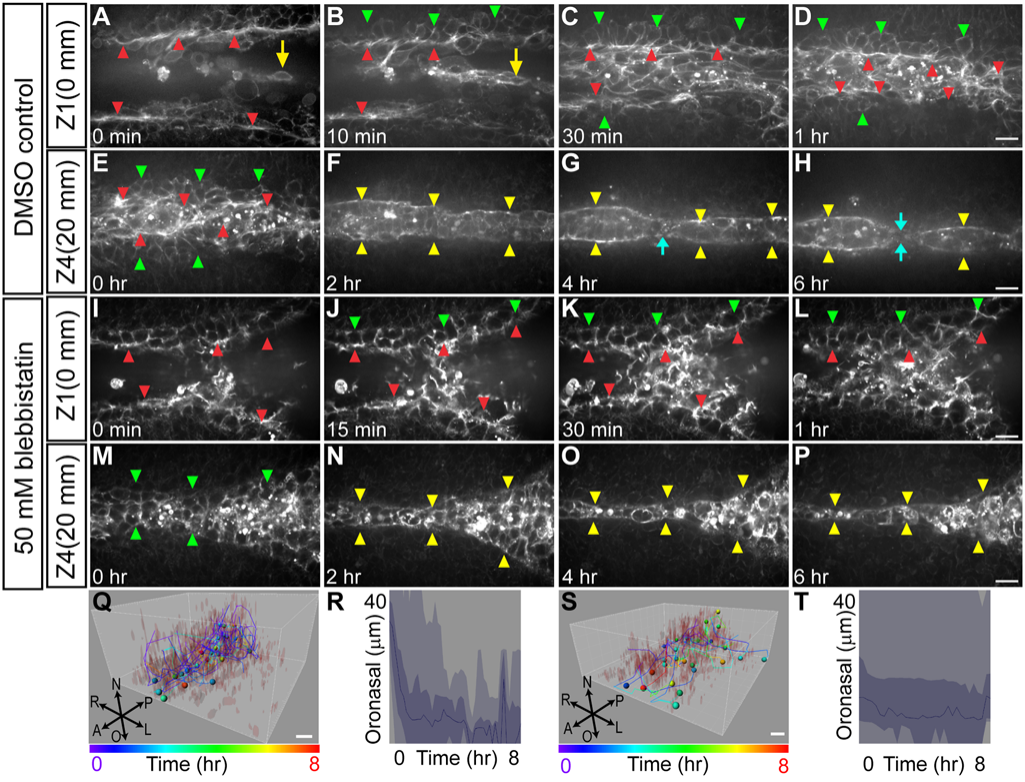
Confocal live imaging of actin cytoskeletal dynamics during palatal fusion. (A–D) Time-lapse imaging of *Lifeact-mRFPruby* palate explant culture reveals prominent multicellular actin cables at the leading edge (red arrowhead) of the palatal shelves as they initiate their closure. In superficial (oral) optical sections (A–D), epithelial cells displaced from deeper layers were observed between the leading edges of the palatal shelves prior to convergence of the shared MES (yellow arrow). The lateral edges of the MES are marked with green arrowheads. (E–H) Imaging at deeper optical sections reveals multicellular actin cables that bound the MES laterally as it broke apart (blue arrows). Scale bar, 20 μm. (I–P) Inhibition of NMII with 50 μM blebbistatin causes defects in epithelial convergence and failure to organize actin cables at the palatal shelf leading edge (red arrowheads) (I–J). (M–P) Imaging of deeper sections shows a thinning and stretched MES, but without lateral actin cables (yellow arrowheads) and the seam did not break as in control. (Q–T) 3-D tracking and vantage plots show oronasal displacement of MES cells in control (Q, R) but not blebbistatin-treated palatal explants (S, T). Scale bar, 10 μm.

Given the requirement for NMII in normal palate fusion, we next examined how filamentous actin was distributed over the course of palatal fusion in relation to the cell behaviors we observed. To observe actin filament dynamics in real time, we imaged explant cultures of *Lifeact-mRFPruby* transgenic mice, which ubiquitously express an RFP-conjugated Lifeact peptide that binds filamentous actin [49,50]. We imaged Lifeact fluorescence over an 8-h period to show actin filament remodeling over the course of initial apposition, formation of a multicellular epithelium, convergence into a single-layered MES, and ultimately, MES breakage. As the palatal shelves approached the midline, multicellular actin cables were observed along the apical edge of the advancing palatal shelves (Fig 5A red arrowheads, S6 Movie). As the palatal shelves moved closer to one another, an abundance of epithelial cells that were rich in filamentous actin were displaced from deeper sections between the edges of the MEE to form a multicellular intermediate structure (Fig 5A, 5B, 5Q and 5R S6 Movie), followed by resolution to an organized MES that was bound laterally by the multicellular actin cable (Fig 5A–5H; S7 Movie). In deeper sections that were undergoing seam breakage, the actin cable on either side of the newly formed MES appeared to contract while the MES pulled apart into separate islands of epithelium and began to dissolve (Fig 5F–5H, S7 Movie). Treating explants with blebbistatin considerably inhibited convergence and oronasal displacement of the MES (Fig 5I–5L, 5S and 5T; S8 Movie), and though the MES was thinner at deeper positions, we did not observe the accumulation of lateral actin cables or breakage of the seam into epithelial islands (Fig 5M–5P; S9 Movie). Treatment of cultures with actin polymerization inhibitors cytochalasin D or latrunculin A had similar effects on the MES to blebbistatin, including loss of a clear leading edge and lateral actin cable structures (S4 Fig).

**Fig 6:**
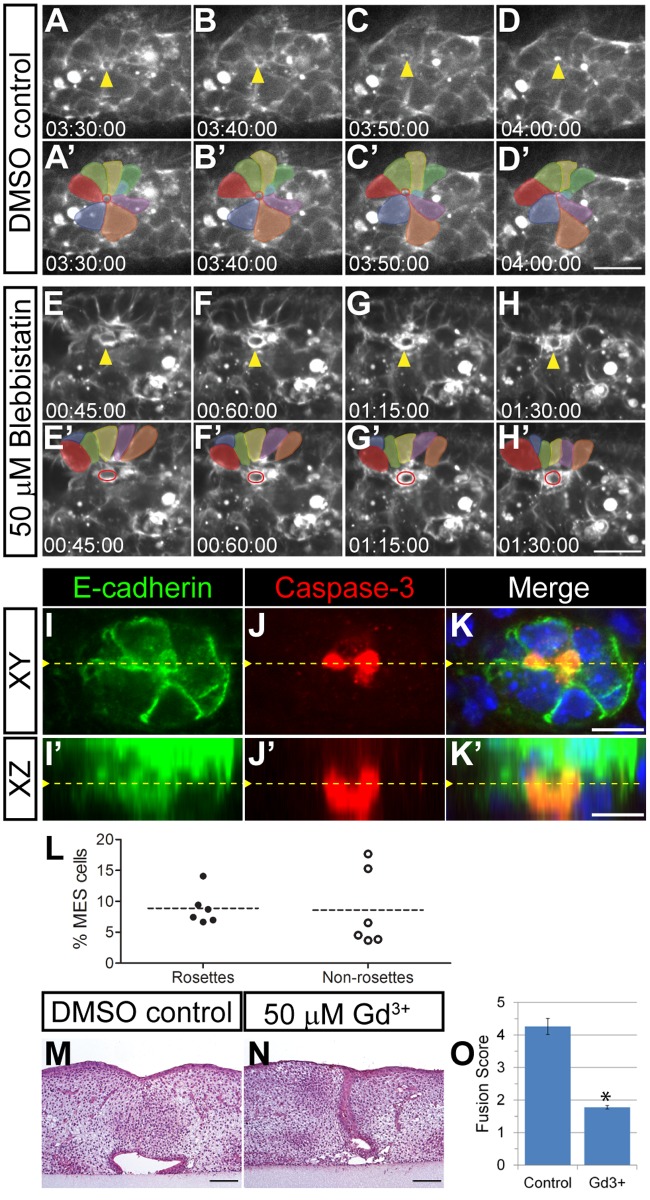
Multi-cellular rosette formation and cell extrusion during secondary palatal fusion. (A–D, A’–D’) An example of rosette formation (colored) and cell extrusion in high magnification confocal live imaging of *Lifeact-mRFPruby* palatal explants. The extruding cell is marked by a yellow arrowhead (A–D) and red circles (A’–D’). Scale bar, 10 μm. (E–H, E’–H’) Disrupted rosette formation is observed upon inhibition of NMII with blebbistatin. The few extrusion-like events that were observed in blebbistatin-treated palate explant cultures did not exhibit well-formed rosettes. Surrounding rosette cells are labeled with different colors. A cell initiating extrusion is indicated by yellow arrowheads (E–H) and red circles (E’–H’). Scale bar, 10 μm. (I–K, I’–K’) Immunostaining for E-cadherin (I, I’) and cleaved caspase-3 (J, J’) indicate that extruding cells are undergoing apoptosis. Orthogonal projection of a confocal Z-stack from which (I–K) are derived shows cell extrusion at the oral side of the MES toward the oral cavity. Yellow dashed line in (I–K) indicates the Y position of (I’–K’); yellow dashed line in (I’–K’) indicates the Z position of (I–K). (L) Percentage of MES cells that are apoptotic. Dotted lines represent the mean of cleaved caspase-3 positive cells as a percentage of total MES cells. (M–O) Treatment with 50 μM gadolinium results in failure of palatal shelf fusion in explants cultured for 72 h (N, O) compared with control (M, O) presented as mean fusion score ± SEM. * *p* < 0.05, Student’s *t* test, *n* = 3. Scale bar, 100 μM. Please see S1 Data for raw data.

Live imaging with *Lifeact-mRFPruby* transgenic mice also revealed striking cell extrusion-like events, in which cellular rosettes surrounded cells with a circular cable of elevated filamentous actin and appeared to squeeze them out of the MES (Fig 6A–6D, 6A’–6D’, S10 Movie) These events greatly resembled cell extrusion that has been reported in other systems, in which an epithelial cell destined for extrusion is surrounded by other epithelial cells which contract an actomyosin ring that squeezes the cell out [51–54]. During normal palatal fusion, these events were frequent, with 101 observed across 16 optical sections of the anterior palate over an 8-h period in two independent live imaging experiments. Treatment with blebbistatin or cytochalasin D reduced the formation of multi-cellular rosette structures, and extrusion events were rarely observed, with 18 seen across 14 optical sections over an 8-h period in two independent live imaging experiments treated with blebbistatin and 20 seen across 12 optical sections over an 8-h period when treated with cytochalasin D. The few extrusion-like events that were observed in blebbistatin-treated palate explant cultures did not exhibit well-formed rosettes (Fig 6E–6H, 6E’–6H’, S11 Movie). To determine whether cell extrusion occurred in intact embryos, and whether extruding cells were undergoing apoptosis, we performed immunostaining of transverse sections of E14.5 embryos for cleaved caspase-3 and E-cadherin (Fig 6I–6K’). Across six embryos, we found that approximately 8.9 ± 1.12% (mean ± SEM) of MES cells that were apoptotic were also part of a rosette. We found approximately the same proportion of apoptotic cells not part of rosettes (8.6 ± 2.54%), suggesting that apoptotic cell extrusion occurs with similar frequency to extrusion-independent apoptosis (Fig 6L). Although static imaging was not able to determine whether these cells underwent apoptosis during or after extrusion, it does indicate a relationship between cleaved caspase-3 positive cells and multicellular rosettes during palatal fusion in intact embryos. In addition to apoptotic cell extrusion, live cell extrusion can be induced by increased density and crowding force dependent on the activity of stretch-activated ion channels such as Piezo1 [51]. To test whether such a mechanism is at play during secondary palate fusion, we performed explant culture in the presence of gadolinium (Gd^3+^), an inhibitor of stretch-activated ion channels [51,55,56]. Treatment with 50 μM Gd^3+^ blocked palate fusion (mean fusion score = 1.78) compared with control (mean fusion score = 4.26), suggesting that live cell extrusion may play a key role in this process (Fig 6M–6O). Together, these data indicate that supracellular actin cable structures act concomitantly with cell convergence and displacement behaviors during secondary palate fusion and suggest that cellular extrusion-like behavior may contribute to removal of MES cells.

## Discussion

**Fig 7:**
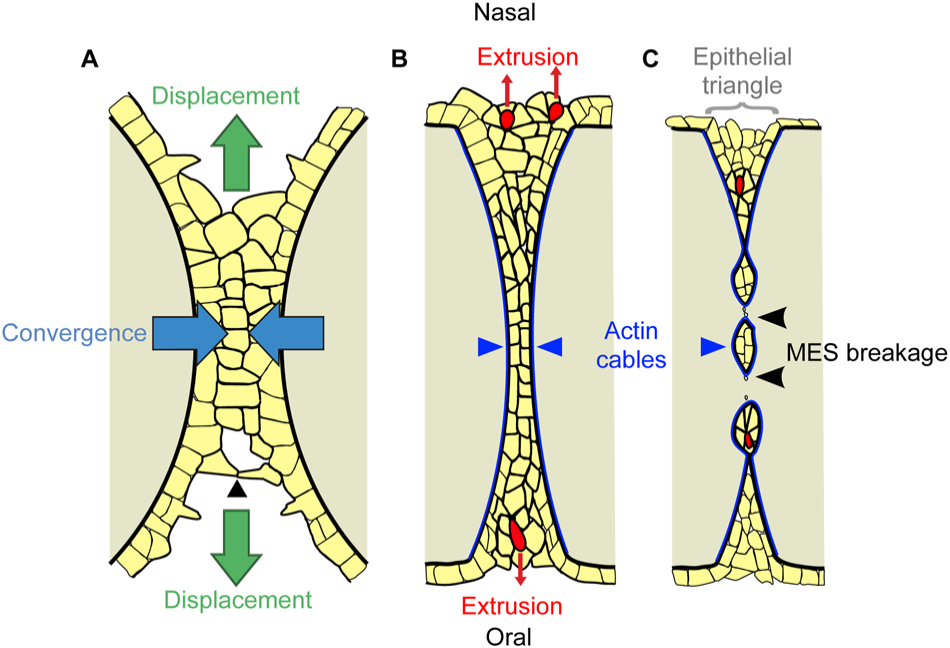
Model of cellular dynamics in palatal fusion. (A–C) Schematic diagram of frontal sections of the MES at progressive stages of palatal fusion. Yellow represents epithelium, tan represents mesenchyme, and red represents extruding cells. (A) Palatal fusion is initiated by cellular protrusions reaching across to form junctions with the apposing shelf (arrowhead). Convergence of the multilayered epithelium toward the midline (blue arrows) is concurrent with oral and nasal displacement of MES epithelium (green arrows). (B) Multicellular actin cables (dark blue) form laterally around the seam as it intercalates to a common MES structure. Formation of multicellular rosettes and cell extrusion (red cells) releases MES cells to the oral and nasal surfaces of the palate. (C) The multicellular actin cables contract, contributing to MES breakage (black arrowheads), and cell extrusion events in the epithelial triangles (grey bracket) continue to remove epithelial cells from the MES.

Here, we identify dynamic cell behaviors underlying mammalian tissue fusion by combining direct observation by live imaging with functional ex vivo and genetic in vivo experiments. Our findings point to a new model for tissue fusion in the secondary palate (Fig 7). Initiation of palatal fusion involves cellular protrusions that establish contacts with the opposite shelf and cellular bridges that eventually give rise to complete contact of the palatal shelves. Recently, live imaging of neural tube closure in mouse embryos revealed that the midbrain neural tube does not close by simple zippering, as in the hindbrain, but rather by a “buttoning” action, where initial contacts lead to intermediate closure points followed by zippering to close the gap between these contacts [57]. Our data indicate that initiation of palatal shelf fusion may be similar to what occurs in the midbrain, with the establishment of transient epithelial bridges at intervals along the fusion front that subsequently zipper closed. In the secondary palate, these bridges form a transient, multi-layered MES structure in a process that involves the displacement of MES cells from deeper positions in the fusing palatal shelves. This multi-layered structure is then integrated toward the midline by intercalation involving cell neighbor exchange. Intercalation is concurrent with displacement of MES cells in the oronasal axis. Blocking convergence by disrupting actomyosin contractility also resulted in failed oronasal MES displacement, suggesting a convergent displacement mechanism for the initiation of MES clearance. Convergence of the MES requires force generated by NMHCIIA, as well as upstream regulators of actomyosin contractility including ROCK, MLCK, and the RLC encoded by the *Myl9* gene. We conclude that convergence force is intrinsic to the epithelium, because loss of NMHCIIA function specifically in this cell type resulted in a failure of MES integration. Our current data do not, however, allow us to determine whether a contribution is made to convergence by non-MES epithelium; a recent report indicates that secondary palate elongation is driven in part by vertical intercalation in the non-MES palate epithelium, suggesting the possibility that convergence forces could in part be generated outside of the MES [58]. As the shared MES begins to dissolve, we observed the lateral accumulation of filamentous actin into multicellular cables, which surrounded and appeared to contract during MES breakage, and NMII-generated contractility was required for MES breakage into epithelial islands.

The involvement of NMHCIIA in palate development takes on particular significance in light of multiple reports that the *MYH9* gene locus is associated with non-syndromic cleft lip and palate in humans [42,59–62]. Although loss of NMHCIIA alone in the palatal epithelium did not lead to a cleft secondary palate in mice, compound disruption of NMHCIIA and NMHCIIB did result in a submucous cleft in the posterior palate. Our analysis clearly supports the involvement of non-muscle myosins in the fusion stage of secondary palate development, which may contribute to its role in human clefting.

Live imaging revealed abundant cell extrusion events characterized by the formation of multi-cellular rosettes surrounding an actin ring and subsequent constriction and squeezing-out of epithelial cells. Cell extrusion can occur by apoptotic or live cell mechanisms [63]. In apoptotic cell extrusion, a cell undergoing apoptosis signals to its neighbors early in the apoptotic process, leading to formation and contraction of an actomyosin ring and squeezing out of the apoptotic cell [53]. In live cell extrusion, epithelial overcrowding induces extrusion of cells that are viable but may then undergo anoikis due to loss of attachment [63]. Our results inhibiting stretch-activated channels indicate that crowding-induced cell extrusion is required for palate fusion. Whether cell extrusion in the MES occurs mainly by apoptotic or live-cell mechanisms remains a question for future study. Regardless, the involvement of cell extrusion in palate fusion might explain the apparent lack of requirement for caspase-mediated apoptosis in removal of the MES because blocking caspase activity had no effect on apoptotic or live cell extrusion [21–23,51,53]. We propose that epithelial cells are ultimately extruded to the oral and nasal surfaces, giving rise to the “epithelial triangles” that have been previously described to contain an elevated number of dying cells (Fig 7C) [11]. Interestingly, early ultrastructural studies are consistent with this model. In transmission electron microscopy studies of rat palatogenesis, Shüpbach et al. observed a process termed “cell exfoliation” and documented the appearance of cell blebs “apparently erupting” from the epithelial triangles of the MES into the oral or nasal cavity [64,65]. More recently, the anterior palate has been described to exhibit a cobblestone-like appearance that depends on *Runx1* expression and correlates with fusion competence, and it will be interesting to learn whether this appearance is related to cell extrusion [66].

Though cell extrusion has been typically observed to regulate cellular homeostasis in a proliferating epithelium, this mechanism has also been shown to be required for epithelial morphogenesis during *Drosophila* dorsal closure [54]. In this case, apoptotic cell extrusion and resultant contraction of the amnioserosa epithelial sheet promotes dorsal closure by pulling epithelial sheets together [54]. The fact that inhibition of apoptosis in explant culture does not interfere significantly with the thinning of the MES suggests it is not likely that apoptosis is the only force driving convergence [20]. Though we are not able to definitively determine the relative contributions of cell extrusion, extrusion-independent apoptosis, and epithelial displacement by convergence to removal of the MES, we did observe that apoptotic cells were at least as commonly part of rosette-like structures as not, indicating that cell extrusion-driven cell death is at least as significant a contributor as extrusion-independent apoptosis. This is likely to be an underestimate of the role of extrusion in fusion, because static quantification may misidentify some cleaved caspase-3 positive cells as not associated with extrusion because (1) the rosette may already have resolved or (2) our inability to observe a rosette not aligned with the plane of optical section. In addition, this calculation does not consider live cell extrusion, which we have shown is also likely to be involved. It is notable that in many systems cellular overcrowding induces cell extrusion; during fusion of the secondary palate, the contact and integration of two palatal shelves results in a doubling of cell density. Furthermore, when cultured in amniotic fluid, palatal shelf epithelium was not removed unless contact was made between the shelves [67], and the anterior secondary palate required contact for initiation of cell death [20]. Consistent with the idea that MES removal is coupled to cell density, culturing with an inhibitor of stretch-activated channels resulted in failed palate fusion, supporting a requirement for crowding-induced extrusion in palate fusion. Taken together, our findings support a novel mechanism of tissue fusion that involves actomyosin-driven convergence, oronasal cell displacement, and cell extrusion.

## Materials and Methods

### Animals

The animal experiments were performed in accordance with the protocols of the University of California at San Francisco Institutional Animal Care and Use Committee. *Myh9* ^*lox/lox*^ (Myh9^tm5Rsad^, MGI ID: 4838521) and *Myh10* ^*lox/lox*^ (Myh10^tm7Rsad^, MGI ID: 4443039) mice have been reported previously [68,69] and were maintained in a 129/Sv and C57BL/6J mixed genetic background. *K14-Cre* (Tg(KRT14-Cre)1Amc, MGI ID: 2445832) [37], *Tgfβ3* ^*cre*^ (Tgfb3tm1(cre)Vk, MGI ID: 3768673) [45], *ROSA26* ^*mTmG*^ (Gt(ROSA)^26Sortm4(ActB-TdTomato,-EGFP)Luo^, MGI ID: 3716464) [70], and *Lifeact-mRFPruby* (Tg(CAG-mRuby)#Rows, MGI ID 4831038) [50] mice were each maintained on a C57Bl/6J coisogenic genetic background.

### Live Imaging

Confocal live imaging of explant cultures was used to visualize palate fusion. Culture media (DMEM/F12 + 20% fetal bovine serum [FBS] + 2mM L-glutamine + 100U/ml Penicillin/100μg/ml Streptomycin + 200μg/ml L-ascorbic acid) was pre-warmed at 37°C. Recently-adhered E14.5 secondary palatal shelves were dissected in culture media and then placed oral surface down in a glass bottom dish (MatTek) with media containing 0.6% low-melting agarose. The dish cover was sealed with petroleum jelly to prevent evaporation of the media. After the culture media was semi-solidified, the culture dish was mounted on a Leica TCS White light SP5 confocal microscope equipped with a 37°C chamber (Experiments Figs 1 and 4; S1–S5 Movie) or a Zeiss Cell Observer spinning disk confocal microscope (Experiments Figs 5 and 6, S4 Fig; S6–S11 Movie). Time-lapse images were captured with 488 nm and 561 nm laser excitation at the indicated intervals. 10 μM Y27632 (Cayman chemical), 50 μM blebbistatin (Sigma), 6 μM cytochalasin D (Sigma), or 2 μM latrunculin A (Cayman chemical) was added to media before imaging.

### Live Imaging Data Analysis

Multiple Z-stack images were obtained using Leica LAS AF software (for the Leica microscope; Figs 1 and 4; S1–S5 Movie) or Zeiss Zen software (for the Zeiss microscope; Figs 5 and 6, S4 Fig; S6–11 Movie). The original live imaging files (.lif or. czi) were opened in Imaris image processing software (Bitplane) for analysis. Bleaching and attenuation correction were performed and signal levels were adjusted in entire movies. Three-dimensional and time crop functions were used to narrow down the region and time window of interest. For cell tracking (Figs 1, 4 and 5; S1 Fig), eGFP, tdTomato, and mRFPruby signals were segmented to generate 3-D membrane surfaces. To identify the center points of the cells, surface signals were masked and inverted images were created. Spot detection found each cell based on these signals, and movements of the spots were traced with autoregressive motion algorithms (S1 Fig) [71]. To visualize cell intercalation in a single plane, images and traces were projected to the XY dimension. Quantification graphs for anteroposterior (X), mediolateral (Y), and oronasal (Z) cell movements were created as Imaris vantage plots. For S3 Movie, surfaces were generated manually in a series of images, and displacement of the center points was traced. In 3-D tracking and 2-D (XY) projections, color-coded spots were inserted to indicate the time points when the spot and trace disappeared. For the middle palate images in S4 Movie, the MtrackJ (ImageJ) plugin was used to track cell migration [72]. To generate rose diagrams in Figs 1 and 4, the displacement angles of traced epithelial and mesenchymal cells were calculated relative to the midline using Imaris software and were plotted using Rose.Net software. For the junctional rearrangements, horizontal and vertical cell junction numbers were counted in seven optical Z sections. A junctional line was drawn over the image and the angle relative to the midline was measured using ImageJ. Junctions between -30º and 30º were considered as horizontal junctions. Junctions between 60º and 120º were counted as vertical junctions. Cell distances between randomly selected pairs of neighboring cells in the same Z section were measured at each time point. Cell intercalation events by neighbor cell exchange were counted in seven Z sections. In Fig 6, cell extrusion events were measured in both live imaging and static immunostained MES sections. Caspase3-positive cells were counted to examine how many cells are undergoing apoptosis in the rosette structure.

### Static Palate Explant Culture

Nitrocellulose filters (Millipore) were placed on triangular metal grids mounted in 6 cm center well culture dishes (Corning). A pair of secondary palatal shelves was dissected at E13.5 and placed in close proximity on the filter paper, which was submerged in culture media (BGjb + 100 U/ml Penicillin/100 μg/ml Streptomycin + 200 μg/ml L-ascorbic acid) with the oral side upward. The center well dish was placed inside of a 10 cm dish on top of gauze soaked with water to maintain humidity. DMSO, 10 μM blebbistatin (Sigma), 10 μM Y27632 (Cayman chemical), 50 μM ML-7 (Calbiochem), or 50 μM GdCl_3_ was added to the media. Explants were cultured at 37°C with 5% CO_2_ for 72 h for fusion analysis or 48 h for immunostaining. Culture media were replaced every day. Explants were transfected with pools of four siRNA sequences at 500 nM for *Myh9*, *Myl9*, or a pool of four scrambled RNAs (ON-TARGETplus SMARTpool, Thermo Scientific) using Lipofectamine 2,000 (Life Technology) diluted in BGjb media to knockdown gene expression. Target sequences were as follows Myh9-05: AAAUUCAUUCGUAUCAACU, Myh9-06: GAGGCACGAGAUGCCACCC, Myh9-07: UUUGGAAACGCCAAGAGGU, Myh9-08: GUAUCAAUGUGACCGACUU and Myl9-09: GCGACCGAUUCACGGAUGA, Myl9-10: CGAGAUGUACCGCGAGGCA, Myl9-11: CCCAAAGGCAAGAUGUCGA, Myl9-12: AUAAGGAGGACCUGCACGA. Palate explants were cultured with siRNA for 72 h and media was changed every day. Fusion scores were analyzed as previously described [73]. Palate fusion score was scored from 1 (incomplete fusion) to 5 (complete fusion). The score was determined based on how much of the MES cell layer was retained after 72 h in culture as follows: Score 1 = A complete multi-cell layer of MES cells remained between two palatal shelves; Score 2 = A mostly continuous MES layer is persistent in the midline of the seam with some regions of a single-cell layer and few breaks; Score 3 = Sections exhibit around 50% removal of the MES; Score 4 = Few MES islands are present; Score 5 = Fusion is complete, with no MES cells remaining. SiRNA knockdown was verified by quantitative real time (qRT)-PCR using the BioRad CFX 96 Real-Time PCR detection system. *Gapdh* was used as a reference gene to normalize the level of expression. QRT-PCR primer sequences were as follows: *Myh9*, Forward 5’-GGCCCTGCTAGATGAGGAGT-3’ and Reverse 5’-CCGGCATAGTGGATAATGCAGA-3’; *Myl9*, Forward 5’-ACAGCGCCGAGGACTTTTC-3’ and Reverse 5’-AGACATTGGACGTAGCCCTCT-3’; *Gapdh*, Forward 5’-CACTGAGCATCTCCCTCACA-3’ and Reverse 5’-TGGGTGCAGCGAACTTTATT-3’.

### Histology

Embryos were dissected at specified stages and fixed in Bouin’s solution for at least 24 h. Embryos were dehydrated through a graded series of ethanol, embedded in paraffin and sectioned at 7 μm thickness prior to staining with hematoxylin and eosin. Fusion score counting was performed using the same criteria as in explant fusion assay [73].

### In Situ Hybridization and Immunostaining

Embryos were fixed with 4% paraformaldehyde in phosphate-buffered saline (PBS, pH 7.4) overnight and submerged in 12.5%, 25% sucrose, and 25% sucrose/OCT solutions sequentially. Fixed embryos were embedded in OCT compound (Tissue-Tek) and sectioned at 12 μm thickness using a cryostat (Fisher Scientific). Tissue slides were kept at -20°C. In situ hybridization was performed with digoxygenin-labeled *Myh9*, *Myh10*, *Myh14*, and *Myl9* antisense or sense probes according to standard methods. Immunostaining was performed using the following primary antibodies: anti-rabbit NMHCIIA (Covance, 1:500), Phalloidin-conjugated Alexa 488 (1:200), anti-rat E-cadherin (Invitrogen, 1:300), anti-rabbit p63 (Abcam, 1:200), anti-rabbit Ki67 (Thermo Scientific, 1:200), and anti-rabbit cleaved caspase-3 (Asp175) (Cell signaling, 1:200). Anti-rabbit IgG-Cy3, anti-rat Cy2, or Dylight 649 (Jackson Immunoresearch Lab) were used as secondary antibodies to visualize the signals. Images were captured using a Zeiss Axio Imager Z2 microscope or a Zeiss Cell Observer spinning disk confocal microscope.

## Supporting Information

S1 Data Raw data and statistical analyses are presented in spreadsheet format. Data for each figure panel and descriptions are listed under tabs with corresponding figure labels.
(XLSX)

S1 Fig Live imaging of palate explant culture. (A) *K14-cre; ROSA26* ^*mTmG/+*^ palate was dissected at E14.5. When mandible and tongue were removed, the MES between two secondary palatal shelves showed strong eGFP-positive cells. This recently adhered palate was dissected together and positioned with the oral surface facing down in a glass-bottomed dish. Time-lapse imaging was performed for 12–16 h at 37°C with imaging every 10–15 min. Scale bar, 500 μm. (B) A palate explant was cultured for 72 h in live imaging media with low melting agarose to examine whether complete fusion occurs under these conditions. Removal of midline MEE cells was confirmed by hematoxylin and eosin (H&E) staining. Scale bar, 100 μm. (C) A *ROSA26* ^*mTmG/+*^ mouse was crossed with an epithelial-specific *K14-cre* mouse to label palate epithelium. (D) Images were analyzed using Imaris software. To identify the centers of cells in the original anterior palate live imaging data (1), a membrane surface was created based on the epithelial eGFP signals of *K14-cre;ROSA26* ^*mTmG/+*^ palate (2). The membrane surface was masked, and an inverted image was generated (3). The spot function was used to detect the centers of individual cells based on the inverted EGFP signals (4). Scale bar, 20 μm.
(TIF)

S2 Fig NMHCIIA is required for normal palate fusion. (A, D, E, F) *Myh9* mRNA is strongly expressed in the palate epithelium and nasal septum during fusion as detected by an antisense probe (A, D, E) whereas a sense control probe yielded no signal (F). Scaling was not recorded for (A), Scale bar for (D), 1 mm. Scale bar for (E, F), 100 μm. (B) Broad, moderate *Myh10* mRNA expression was observed in the mesenchyme by in situ hybridization. (C) *Myh14* mRNA was not detected. Scale bar, 100 μm. (G–I) NMHCIIA and filamentous actin are strongly expressed in the palate epithelium, including the MEE, at the fusion stage. Scale bar, 100 μm. (J–L) Inhibition of NMII ATPase activity with blebbistatin in explant culture resulted in defects in palate fusion. (M) Cell proliferation in blebbistatin-treated explants quantified by the percentage of Ki67^+^ cells in *n* = 3 explants. (N–P) Knockdown of *Myh9* using siRNA caused defects in fusion in palate explant culture. Scale bar, 100 μm. Immunostaining for NMHCIIA (Q, R) and quantitative RT-PCR (S) confirmed that *Myh9* expression was significantly reduced in the siRNA-treated palate. (T-V) *Myl9* mRNA expression was detected in the mesenchyme and at elevated levels in the palate epithelium with an antisense in situ hybridization probe (T,U), whereas sense control probe yielded no signal (V). Scale bar for (T), 1 mm. Scale bar for (U, V), 100 μm. In L and P, data are presented as mean fusion score ± SEM. * *p* < 0.05, Student’s *t* test, *n* = 7–8 in L, *n* = 3 in P. In R, data are presented as mean relative expression ratio to *Gapdh* ± SEM. * *p* < 0.05, Student’s *t* test, *n* = 4. Please see S1 Data for raw data.
(TIF)

S3 Fig Compound loss of *Myh9* and *Myh10* lead to more severe defects in palate fusion. (A) *K14-cre; Myh9* ^*lox/lox*^ mutants showed defects in palate fusion at E15.5 and retained MES epithelium (arrows in b, d) compared with control (a, c). (B) Mean fusion score was significantly reduced in the anterior and middle palate regions compared with *Myh9* ^*lox/lox*^ control. (C) Cell proliferation rate, as measured by counting Ki67^+^ nuclei as a percentage of DAPI^+^ nuclei (D) NMHCIIA expression was not completely lost in *K14-cre; Myh9* ^*lox/lox*^ mutant palate epithelium at E14.5 (a, b), whereas *Tgfβ3* ^*cre/+*^ mediated nearly complete removal of NMHCIIA (c, d). Scale bar, 100 μm. (E) Fragmented segments of the MES (black arrows in b, d) perdure in the anterior and middle palates of *Tgfβ3* ^*cre/+*^; *Myh9* ^*lox/lox*^ mutant embryos at E17.5 (b, d), but are completely gone from comparable sections of control (a, c). Scale bar, 100 μm. (F) *Tgfβ3* ^*cre/+*^; *Myh10* ^*lox/lox*^ mutant embryos exhibit normal fusion of the secondary palate (b, d, f) compared with control (a, c, e) (G) *Tgfβ3* ^*cre/+*^; *Myh9* ^*lox/lox*^; *Myh10* ^*lox/lox*^ compound mutants show severe defects in palate fusion in all regions at E15.5 (black arrows in b, d). Scale bar, 100 μm. In B, data are presented as mean fusion score ± SEM. * *p* < 0.05, Student’s *t* test, *n* = 3. Please see S1 Data for raw data.
(TIF)

S4 Fig Actin polymerization is required for formation of multicellular cables and proper palate fusion morphogenesis. Time-lapse imaging of *Lifeact-mRFPruby* palatal explants treated with 6 μM cytochalasin D (A-H) or 2 μM latrunculin A (I-P). The position of the medial edge of the palatal shelves is marked with red arrowheads. Green arrowheads mark the lateral boundary of the MES and yellow arrowheads mark the position where lateral actin cables should be forming.
(TIF)

S1 Movie Initiation of palatal fusion and MES convergence. Live imaging of the Z3 (10 μm) plane of a *K14-cre; ROSA26* ^*mTmG/+*^ anterior palate shows convergence of two epithelial layers into a single layer of MEE. Membrane protrusions from epithelial cells were noted in the anterior region before convergence. Scale bar, 20 μm. Images were captured every 10 min for 16 h.
(AVI)

S2 Movie Cellular protrusions connect the apposed palatal shelves. Live imaging of the Z7 (35 μm) plane of the anterior end of the palate, which has not yet converged, shows two epithelial cells from each palatal shelf extending membrane protrusions. Initial contact occurs at a single membrane point, and the junction extends along the anterior-posterior axis as fusion proceeds. Scale bar, 10 μm. Images were captured every 10 min for 8 h and 20 min.
(AVI)

S3 Movie Cell intercalation and neighbor exchange occurs during MES convergence. Manual surface-volume rendering from high magnification imaging of the Z3 (10 μm) section was performed using Imaris software. Individual cells were labeled with the same colors as in Fig 1O–1R. Surface traces were color-coded with time. The red and yellow cells intercalate by rearranging their cellular junctions with neighboring cells. These two cells become aligned in the resulting single-layered MEE. Scale bar, 10 μm. Images were captured every 10 min for 12 h and 30 min.
(AVI)

S4 Movie Occasional antero-posterior migration events occur during palatal fusion. Live imaging of the middle palate region of *K14-Cre; ROSA26* ^*mTmG/+*^ embryos reveals superficial oral cells that migrate posteriorly. Tracking of the midline cells in the Z2 (5 μm depth) plane was performed using ImageJ. Five different cells were labeled with different colors. Circles show the centers of the cells, and lines indicate the traces of cell migration over time. Scale bar, 20 μm. Images were captured every 15 min for 13 h.
(AVI)

S5 Movie ROCK is required for convergence and displacement of the MES. Live imaging of the Z1 (0 μm, oral surface) plane of a *K14-cre; ROSA26* ^*mTmG/+*^ anterior palate explant after treatment with 10 μM Y27632 reveals retarded epithelial cell migration to the midline and failure of cell intercalation. Scale bar, 20 μm. Images were captured every 15 min for 19 h and 35 min.
(AVI)

S6 Movie Dynamic changes in contractile actin are observed during the initiation of fusion and MES convergence. Live imaging of the Z1 (0 μm, oral surface) plane of a *Lifeact-mRFPruby* anterior palate explant culture shows multicellular actin cables between two palate epithelial layers converging to the midline during fusion. The leading edge of the palate shelves are indicated with red arrowheads. The lateral edges of the MES are labeled with green arrowheads. Displacement of epithelial cells from deeper Z levels is prominent at early time points (yellow arrows). Multiple rosette structures and cell extrusion events were observed (red arrows). Scale bar, 20 μm. Images were captured every 10 min for 7 h and 40 min.
(AVI)

S7 Movie Contractile actin accumulates at the lateral edges of the MES during breakage. Live imaging of the Z4 (20 μm, deep) plane of an *Lifeact-mRFPruby* anterior palate explant shows breakage (blue arrow) of the MES prompted by actin contraction, with multiple rosettes and cell extrusion events (red arrow). Scale bar, 20 μm. Images were captured every 10 min for 7 h 40 min.
(AVI)

S8 Movie NMII is required for normal MES convergence. A *Lifeact-mRFPruby* anterior palate explant treated with 50 μM blebbistatin fails to converge to a single MES at the oral surface. The leading edge (red arrowheads) does not accumulate elevated filamentous actin. Green arrowheads indicate the lateral boundaries of the MES. Scale bar, 20 μm. Images were captured every 15 min for 8 h.
(AVI)

S9 Movie NMII is required for normal breakage of the MES. Live imaging of the Z4 (20 μm, deep) plane of a *Lifeact-mRFPruby* anterior palate explant treated with 50 μM blebbistatin. The MES does not fuse, although the seam becomes thinner. Green arrowheads indicate the lateral boundaries of the MES. Scale bar, 20 μm, Images were captured every 15 min for 8 h.
(AVI)

S10 Movie Cell extrusion occurs during palate fusion. Confocal live imaging of the Z2 (5 μm deep) plane of a *Lifeact-mRFPruby* anterior palate explant reveals numerous cell extrusion events (yellow arrowheads) that are often preceded by the formation of multicellular rosette like structures (color highlighted) that surround the cell fated for extrusion. Scale bar, 20 μm. Images were captured every 10 min for 7 h and 40 min.
(AVI)

S11 Movie NMII activity is required for rosette formation and cell extrusion. Confocal live imaging of the Z2 (5 μm deep) plane of a *Lifeact-mRFPruby* anterior palate explant treated with 50 μM blebbistatin reveals the presence of few multicellular rosettes and lack of cell extrusion. Cells undergo membrane ruffling and do not appear to contract the MES. Scale bar, 20 μm. Images were captured every 15 min for 8 h.
(AVI)

## Abbreviations

ELC: essential light chain
EMT: epithelial mesenchymal transition
H&E: hematoxylin and eosin
K14: Keratin 14
MEE: Medial Edge Epithelium
MES: Medial Epithelial Seam
MLCK: Myosin Light Chain Kinase
mTmG: membrane-targeted Tomato membrane-targeted EGFP
MYPT1: Myosin Phosphatase Target subunit 1
NMHC: Non-muscle Myosin Heavy Chain
NMII: Non-muscle Myosin type II
p63: Tumor Protein p63
RLC: Regulatory Light Chain
ROCK: Rho-associated Protein Kinase
Runx1: Runt-related Transcription Factor 1
qRT-PCR: quantitative real time PCR
SEM: Standard Error of the Mean
