# Supplementary figures and images for: Convergence and Extrusion Are Required for Normal Fusion of the Mammalian Secondary Palate
Source: PLoS Biol. 2015 Apr 7;13(4):e1002122. doi: 10.1371/journal.pbio.1002122 (PMC4388528; doi:10.1371/journal.pbio.1002122)

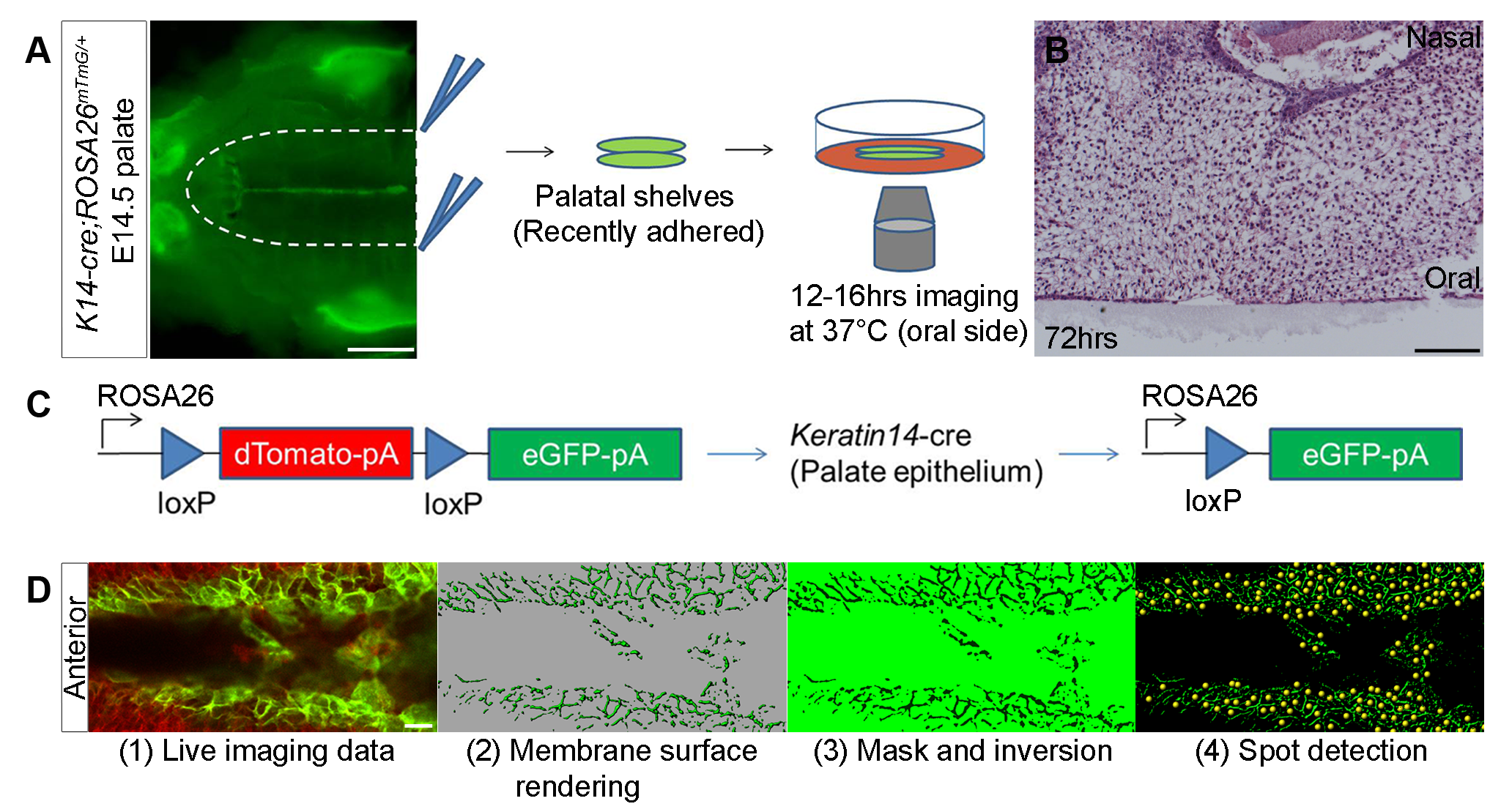

Supplement: S1 Fig — (A) K14-cre; ROSA26 mTmG/+ palate was dissected at E14.5. When mandible and tongue were removed, the MES between two secondary palatal shelves showed strong eGFP-positive cells. This recently adhered palate was dissected together and positioned with the oral surface facing down in a glass-bottomed dish. Time-lapse imaging was performed for 12–16 h at 37°C with imaging every 10–15 min. Scale bar, 500 μm. (B) A palate explant was cultured for 72 h in live imaging media with low melting agarose to examine whether complete fusion occurs under these conditions. Removal of midline MEE cells was confirmed by hematoxylin and eosin (H&E) staining. Scale bar, 100 μm. (C) A ROSA26 mTmG/+ mouse was crossed with an epithelial-specific K14-cre mouse to label palate epithelium. (D) Images were analyzed using Imaris software. To identify the centers of cells in the original anterior palate live imaging data (1), a membrane surface was created based on the epithelial eGFP signals of K14-cre;ROSA26 mTmG/+ palate (2). The membrane surface was masked, and an inverted image was generated (3). The spot function was used to detect the centers of individual cells based on the inverted EGFP signals (4). Scale bar, 20 μm. (TIF) [file pbio.1002122.s002.tif]

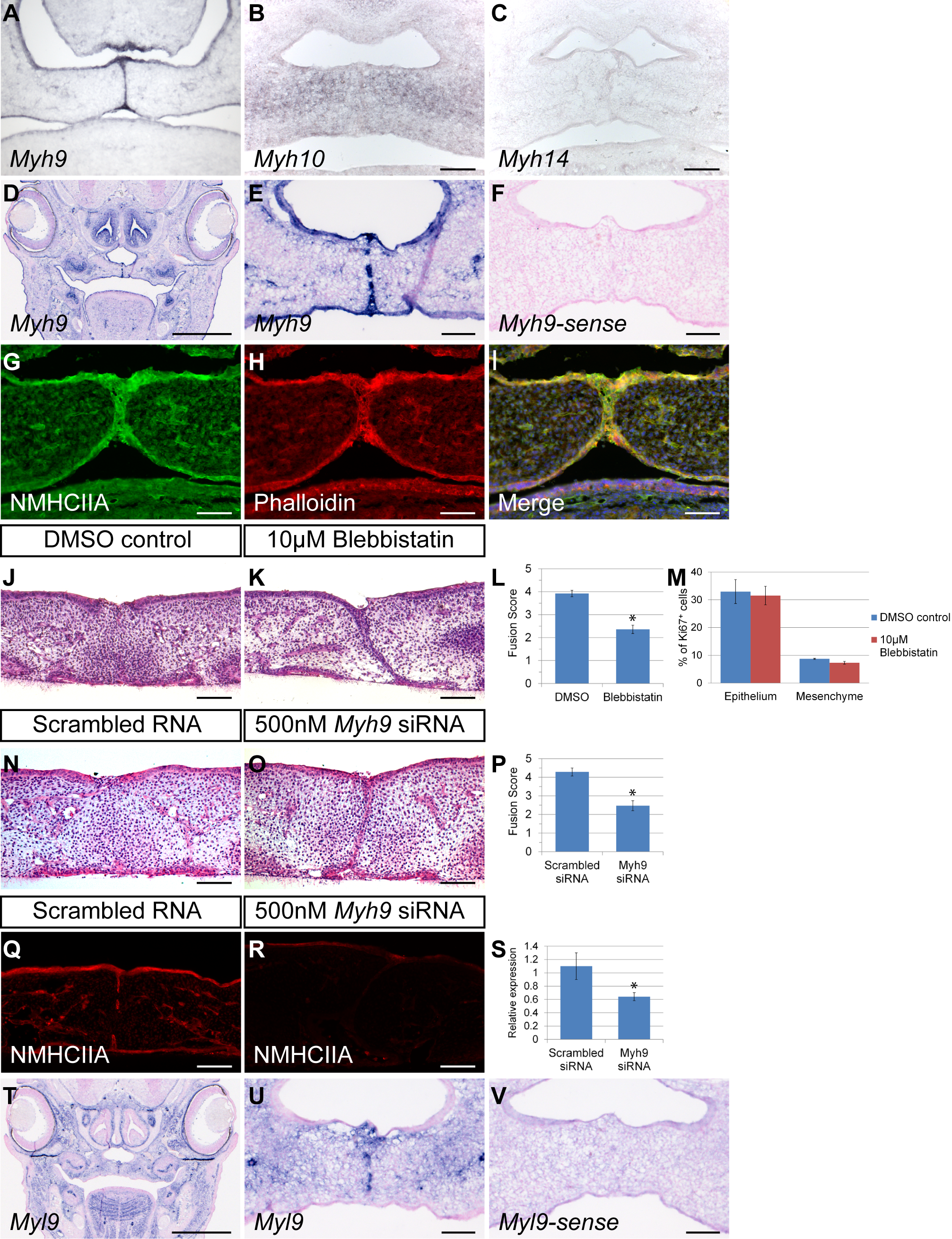

Supplement: S2 Fig — (A, D, E, F) Myh9 mRNA is strongly expressed in the palate epithelium and nasal septum during fusion as detected by an antisense probe (A, D, E) whereas a sense control probe yielded no signal (F). Scaling was not recorded for (A), Scale bar for (D), 1 mm. Scale bar for (E, F), 100 μm. (B) Broad, moderate Myh10 mRNA expression was observed in the mesenchyme by in situ hybridization. (C) Myh14 mRNA was not detected. Scale bar, 100 μm. (G–I) NMHCIIA and filamentous actin are strongly expressed in the palate epithelium, including the MEE, at the fusion stage. Scale bar, 100 μm. (J–L) Inhibition of NMII ATPase activity with blebbistatin in explant culture resulted in defects in palate fusion. (M) Cell proliferation in blebbistatin-treated explants quantified by the percentage of Ki67+ cells in n = 3 explants. (N–P) Knockdown of Myh9 using siRNA caused defects in fusion in palate explant culture. Scale bar, 100 μm. Immunostaining for NMHCIIA (Q, R) and quantitative RT-PCR (S) confirmed that Myh9 expression was significantly reduced in the siRNA-treated palate. (T-V) Myl9 mRNA expression was detected in the mesenchyme and at elevated levels in the palate epithelium with an antisense in situ hybridization probe (T,U), whereas sense control probe yielded no signal (V). Scale bar for (T), 1 mm. Scale bar for (U, V), 100 μm. In L and P, data are presented as mean fusion score ± SEM. * p < 0.05, Student’s t test, n = 7–8 in L, n = 3 in P. In R, data are presented as mean relative expression ratio to Gapdh ± SEM. * p < 0.05, Student’s t test, n = 4. Please see S1 Data for raw data. (TIF) [file pbio.1002122.s003.tif]

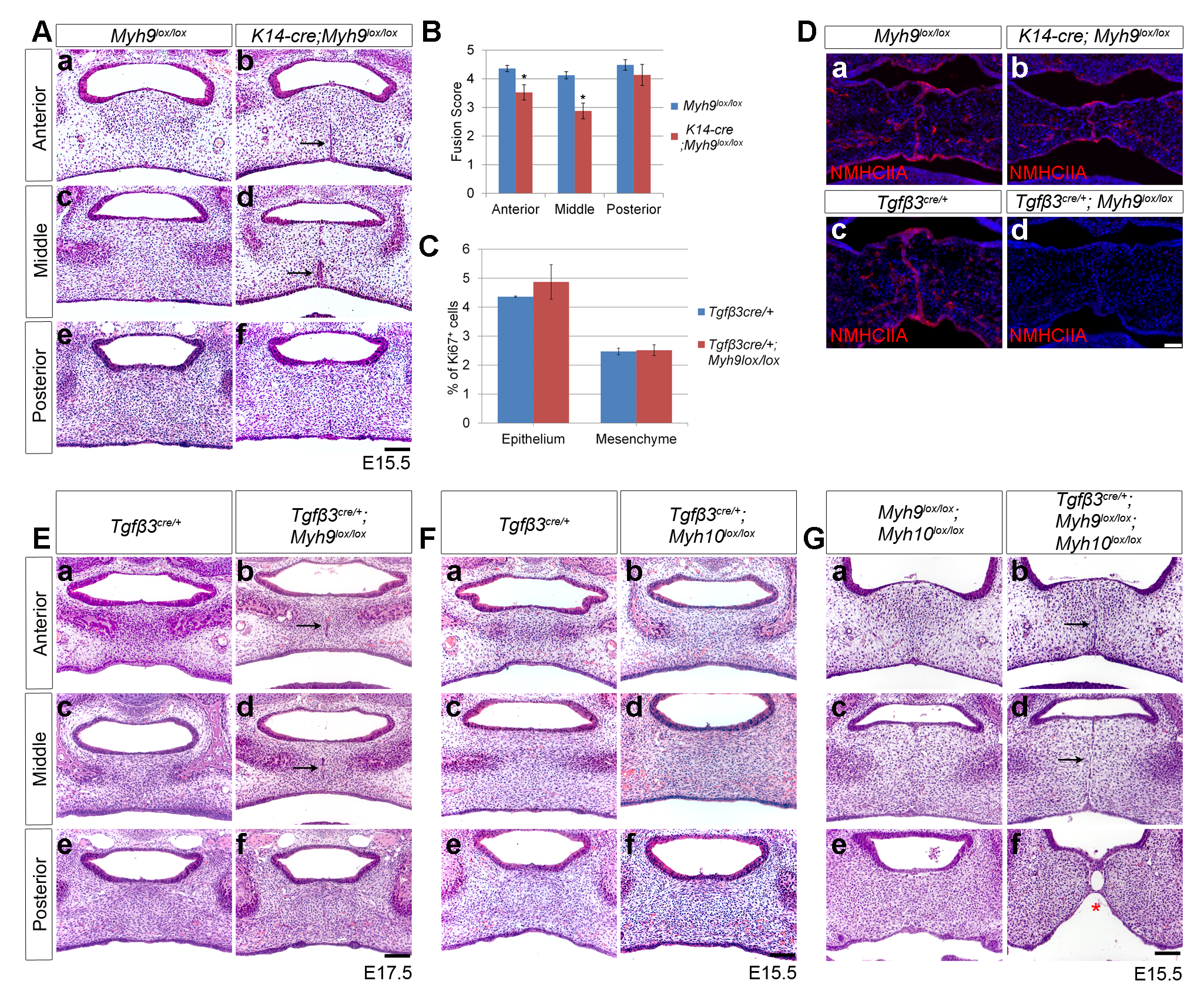

Supplement: S3 Fig — (A) K14-cre; Myh9 lox/lox mutants showed defects in palate fusion at E15.5 and retained MES epithelium (arrows in b, d) compared with control (a, c). (B) Mean fusion score was significantly reduced in the anterior and middle palate regions compared with Myh9 lox/lox control. (C) Cell proliferation rate, as measured by counting Ki67+ nuclei as a percentage of DAPI+ nuclei (D) NMHCIIA expression was not completely lost in K14-cre; Myh9 lox/lox mutant palate epithelium at E14.5 (a, b), whereas Tgfβ3 cre/+ mediated nearly complete removal of NMHCIIA (c, d). Scale bar, 100 μm. (E) Fragmented segments of the MES (black arrows in b, d) perdure in the anterior and middle palates of Tgfβ3 cre/+; Myh9 lox/lox mutant embryos at E17.5 (b, d), but are completely gone from comparable sections of control (a, c). Scale bar, 100 μm. (F) Tgfβ3 cre/+; Myh10 lox/lox mutant embryos exhibit normal fusion of the secondary palate (b, d, f) compared with control (a, c, e) (G) Tgfβ3 cre/+; Myh9 lox/lox; Myh10 lox/lox compound mutants show severe defects in palate fusion in all regions at E15.5 (black arrows in b, d). Scale bar, 100 μm. In B, data are presented as mean fusion score ± SEM. * p < 0.05, Student’s t test, n = 3. Please see S1 Data for raw data. (TIF) [file pbio.1002122.s004.tif]

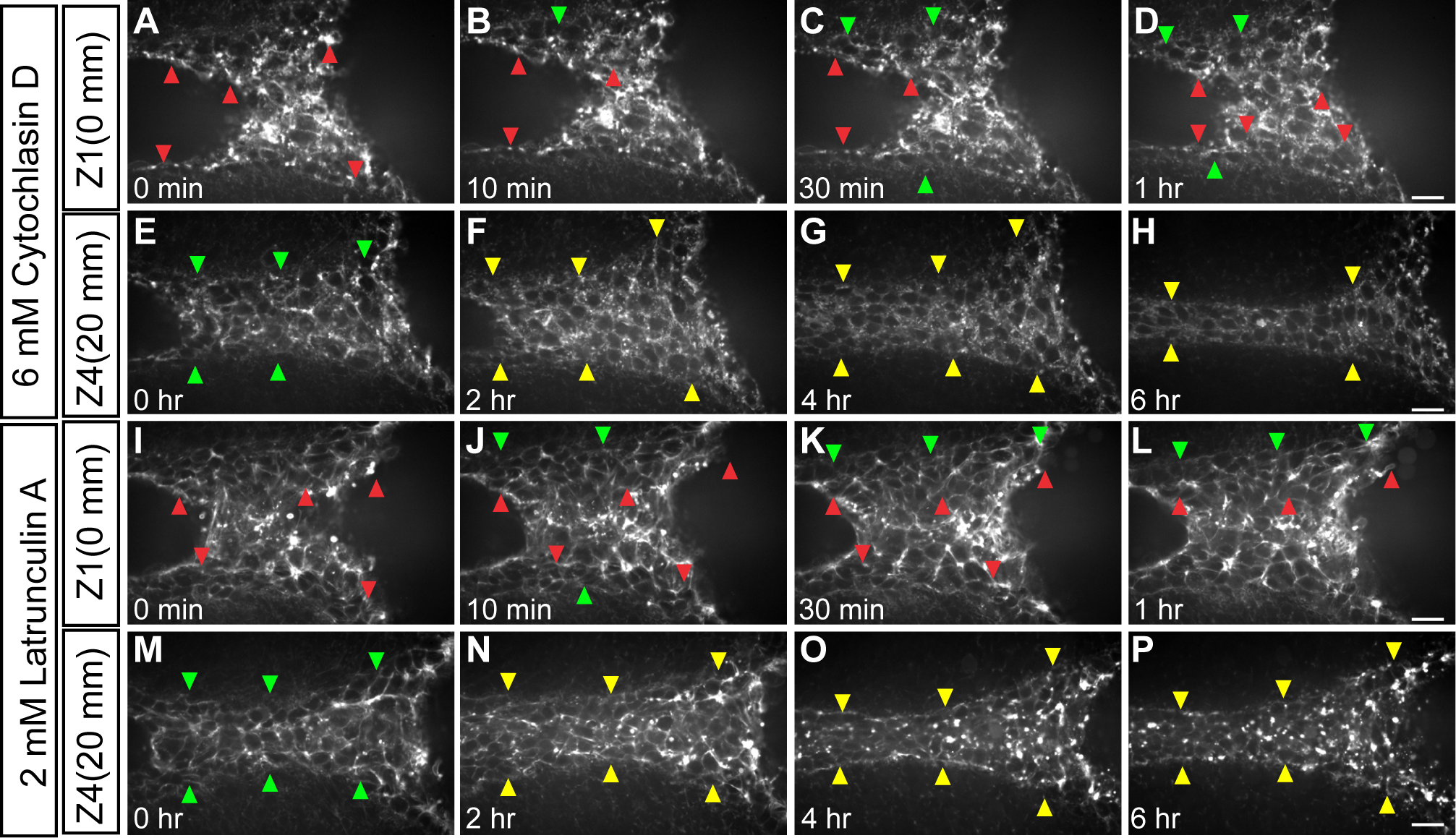

Supplement: S4 Fig — Time-lapse imaging of Lifeact-mRFPruby palatal explants treated with 6 μM cytochalasin D (A-H) or 2 μM latrunculin A (I-P). The position of the medial edge of the palatal shelves is marked with red arrowheads. Green arrowheads mark the lateral boundary of the MES and yellow arrowheads mark the position where lateral actin cables should be forming. (TIF) [file pbio.1002122.s005.tif]
